# Individuals’ Assessments of Their Own Wellbeing, Subjective Welfare, and Good Life: Four Exploratory Studies

**DOI:** 10.3390/ijerph191911919

**Published:** 2022-09-21

**Authors:** Micael Dahlen, Helge Thorbjørnsen

**Affiliations:** 1 Stockholm School of Economics, P.O. Box 6501, SE-113 83 Stockholm, Sweden; 2 Norwegian School of Economics, Helleveien 30, 5045 Bergen, Norway

**Keywords:** wellbeing, subjective welfare, good life, happiness, meaning in life, psychological richness

## Abstract

This paper reports on four exploratory online studies of how wellbeing and welfare are valued and perceived from a subjective, individual perspective. Study 1 (*n* = 707) compares individuals’ subjective ratings and correlations of the importance of the three wellbeing dimensions happiness, meaning in life, and a psychologically rich life, as well as their welfare. Study 2 (*n* = 679) factor-analyses the same four (five-item) wellbeing and subjective welfare constructs. Study 3 (*n* = 710) gauges how individuals’ global assessments of the three dimensions of wellbeing and of subjective welfare contribute to their assessments of living a good life, using stepwise regression analysis. Study 4 (*n* = 663) replicates the stepwise regression analysis with global measures of relative, rather than absolute, wellbeing and subjective welfare.

## 1. Introduction

Would you be able to rate the importance of the dimensions of wellbeing in your life, and of your own welfare? How would the assessments of your wellbeing and welfare relate and contribute to your experience of living a good life? These are the questions we seek to answer in four exploratory studies. While the studies of wellbeing and welfare are both subject to significant bodies of literature, dating back more than a century, there is a dearth of research into their relationships on an individual, subjective level. There are several reasons why, we believe, the relationships between wellbeing and welfare merit further study.

First, wellbeing and welfare are simultaneously gaining increasing and explicit focus in public policy. For example, they are both high on the United Nations 2030 agenda, which is signed by all member states [1]. Wellbeing is listed as the third of 17 sustainable development goals, and welfare is arguably represented in several of the goals (e.g., in terms of economy, good health, and inclusiveness).

Second, while ideally, both would be given maximum priority, policymakers and individuals may increasingly find themselves in situations where they must trade off one for the other [2]. Trading off decreasing wellbeing for maintained welfare in terms of health and economic security, through quarantines and short-term layoffs during the COVID pandemic, would be one such example [3,4]. Another example would be a conflict between ecological welfare interventions, to ultimately preserve or enhance living conditions, and decreasing people’s wellbeing from reduced consumption [5].

Third, wellbeing and welfare are seemingly subject to conceptual confusion. For example, some view wellbeing as a subdimension of welfare [6,7], while others treat welfare as a (potential) antecedent to wellbeing [8,9,10]. Moreover, welfare and wellbeing have been viewed as being on a macro level versus individual level, respectively [11,12,13], and alternately, wellbeing has been used as a measure of subjective welfare [14,15,16].

In this paper, we explore the possibility of gauging welfare in a similar, subjective individual-level manner as wellbeing, and of conceptualizing it as a fourth dimension, alongside the three dimensions of wellbeing; happiness, meaning in life, and psychological richness [17], that potentially contribute to an individual’s perception of living a good life. To this end, we conducted four surveys of Swedes recruited on Facebook (*n*_1_ = 707, *n*_2_ = 679, *n*_3_ = 710, *n*_4_ = 663).

## 2. Three Dimensions of Wellbeing

Wellbeing is subject to a massive body of research, dating back more than a century and receiving exploding interest in the last ten years. For a review, see [18]. In the last few decades, researchers have seemingly reached consensus around its two main dimensions [19], being subjective wellbeing, in terms of *happiness* (alternative terms that are also used include hedonia and life satisfaction), and psychological wellbeing, in terms of *meaning in life* (alternative terms that are also used include eudaimonia and meaningfulness). These dimensions are conceptualized as an individual’s assessments of how well life is going, and have been found to have stable psychometric properties across measures, methods, time and demographics. For a review, see [20]. In other words, it is proven that people, anywhere and at anytime, are able to rate their own happiness and meaning in life, consistently and without confusion.

In recent years, psychological richness has been proposed as a third dimension alongside happiness and meaning in life, which is an assessment of life as rich in experiences, eventful, interesting, and perspective-shifting [21,22]. A global study shows that people from different parts of the world are indeed able to assess such a dimension of their lives in a similar fashion [17]. Psychometric analysis shows that when taken together, psychological richness contributes uniquely and significantly to people’s wellbeing assessments alongside happiness and meaning in life [22]. We will use these three dimensions for our purpose of relating wellbeing and subjective welfare.

## 3. Subjective Welfare

Welfare is subject to a body of research that dates back even farther than that of wellbeing, and it is far more diverse in terms of dimensions, conceptualization, and measurement. Not surprisingly, a comprehensive review of the research literature is hard to find. However, as it relates to individuals’ wellbeing and the focus of this paper, we find that welfare research can be organized into three distinct streams.

The first, main, stream centers on manifest variables, such as money (in terms of income or economic security), health, housing, and employment. For an overview, see, for example [23]. Research in recent years shows that individuals’ assessments of their own welfare in these terms may differ significantly over time, between socioeconomic cohorts, and even within the same cohorts. For example, surveys of individuals in both developed and developing countries found that their own assessments of their relative income were not very good predictors of their poverty or wealth [14,24]. Similarly, while objectively on the same level, individuals’ assessments of their health have been found to differ between countries [23] and before versus after the COVID pandemic [25]. Even housing needs have been found to differ in manifest versus subjective terms over time [26].

The second stream of welfare research centers on individuals’ assessments of their own welfare, using their wellbeing ratings as measures “subjective welfare” [15,16]. As mentioned in the introduction, this conceptual confusion is a major reason why this paper seeks to find an alternative measure of subjective welfare alongside (rather than substituted by) wellbeing.

The third research stream also centers on individuals’ assessments of their own welfare, but instead uses ratings of capabilities or opportunities to attain welfare. This would include Sen’s classic capability approach [27] to welfare. It gauges subjective wellbeing capabilities, that is, individuals’ assessments of their capabilities to achieve wellbeing through, for example, having or being able to attain economic security, good health, and employment [28,29]. It would also include the needs-and-wants framework, which does not define welfare as a precursor to wellbeing, but instead puts them in parallel, with needs and wants for welfare (similar to the capabilities approach) and wellbeing, respectively [30,31].

We would argue that this latter view of welfare from an individual’s subjective perspective is the most akin to how wellbeing is conceptualized. Research has well established that individuals are able to make similar multi-item scale assessments of their wellbeing. However, wellbeing research has also found that individuals are able to explicitly rate their lives in terms of global happiness, meaningfulness and psychological richness, for example, from 1–10 [5,22]. To the best of our knowledge, it has not yet been established whether they would also be able to explicitly rate their welfare in a similar fashion. A primary goal of this paper is to explore how individuals would rate their own welfare in explicit terms and how this relates to the three different dimensions of wellbeing.

## 4. A Good Life

In our aim to explore whether individuals would be able to rate the importance of the dimensions of wellbeing and of welfare in their lives, and to rate them all explicitly, we turn to the concept of a good life. It dates back to ancient philosophy and the conceptions of Plato and Aristotle of what makes life worth living, and what is a life well lived. For an account of the philosophy, see, for example, Paul et al. [32].

The concept of a good life has been discussed by scholars of both wellbeing and welfare. For example, the good life has been suggested as a higher, ultimate goal beyond wellbeing [33,34], and a super-concept under which the main (material and immaterial) qualities of life could be sorted [35]. In these veins, qualitative studies have investigated people’s notions of a good life [36] and compared them cross-culturally [37].

The notion of a good life has also been used to compare the rated importance of happiness, meaning in life and psychological richness cross-culturally [22], and to make global comparisons of subjective wellbeing and objective welfare measures [38]. We believe that the notion of a good life would also apply well to investigating individuals’ assessments of their own wellbeing and welfare and how they relate to each other, which is the purpose of this paper.

While the good life has been subject to conceptual and qualitative analysis, to the best of our knowledge, only two studies have actually measured it, quantitatively. In those studies, it was operationalized as a two-dimensional measure consisting of desirability and moral goodness [39,40]. For the purpose of this paper, we will use a one-dimensional measure, where individuals rate their own life on a scale 1–10 in terms of the items “a good life”, “a life well-lived so far”, “a life worth living”, and “a life I would wish for others”.

## 5. Study 1: How Do People Rank the Importance of Wellbeing and Subjective Welfare to Their Own Lives?

In this study, we explored how people rate the importance of the three wellbeing dimensions happiness, meaning in life, and psychological richness, and of welfare, to their lives on a 1–10 scale.

### 5.1. Materials and Methods

A total of 707 Swedes (51% females, age span 18–70) were recruited via Facebook ads for a survey of wellbeing and welfare. For database reasons, we were not able to collect personal demographic information. Respondents were informed that they agreed to be included in the study by answering the web-based questionnaire. The study was conducted in accordance with the Declaration of Helsinki [41].

Respondents answered the question, “how important is it that your life is…” on a scale from 1 (not at all important)–10 (very important), with four items (happiness) “happy, pleasant, stable, simple and comfortable”, (meaning in life) “meaningful, purposeful, fulfilling, virtuous and dedicated”, (psychological richness) “psychologically rich, eventful, interesting, dramatic and surprising”, and (subjective welfare) “welfare, economic security, capability for good health, access to what I need, opportunity to do what I want”. The adjectives for the three wellbeing items were taken from Oishi et al. [22], and the adjectives for subjective welfare were derived and combined from the capabilities measures in Anand et al. [28] and needs and wants from Allardt [30]. The questionnaire was in Swedish (in which the terms wellbeing and welfare have the same meaning).

### 5.2. Results

Mean values are reported in Table 1. The three wellbeing dimensions and subjective welfare all rated significantly above the midpoint of the scale (*p* < 0.001). Subjective welfare received the highest mean rating, *M* = 8.24, significantly higher than happiness at *M* = 7.68. Meaning in life rated third (and significantly lower than happiness at *M* = 7.36. Psychological richness rated significantly lowest at *M* = 6.46.

There were weak to moderate bivariate correlations between all the dimensions, except for happiness and psychological richness (*r* = 0.052, *p* = 0.17). Subjective welfare correlated the highest with happiness (*r* = 0.39, *p* < 0.001), and the lowest with psychological richness (*r* = 0.14, *p* < 0.001).

### 5.3. Discussion

The mean ratings suggest that people consider both wellbeing and subjective welfare as important to their own lives. The ranking of the three wellbeing dimensions is similar to the order in which people chose one over the other in Oishi et al.’s global study [22]. However, subjective welfare ranks even higher. The bivariate correlations suggest that the wellbeing and subjective welfare measures overlap to some extent, but mainly tap into unique constructs. However, the single-item combinations of adjectives do not allow for any further analysis of their nomological relationships. This is the focus of Study 2.

## 6. Study 2: Factor Analysis of the Three Dimensions of Wellbeing and Subjective Welfare

In this study, we factor analyzed people’s ratings of (4 × 5) items for happiness, meaning in life, psychological richness, and subjective welfare to see how they relate.

### 6.1. Materials and Methods

A total of 679 Swedes (50% females, age span 18–70) were recruited via Facebook ads for a survey of wellbeing and welfare. They received the same information as in Study 1.

Respondents answered the question, “how would you rate your life in terms of…” on a scale from 1 (very low)–10 (very high), with 20 items. The items were identical to the adjectives in Study 1: (happiness) “happy”, “pleasant”, “stable”, “simple” and “comfortable”, (meaning in life) “meaningful”, “purposeful”, “fulfilling”, “virtuous” and “dedicated”, (psychological richness) “psychologically rich”, “eventful”, “interesting”, “dramatic” and “surprising”, and (subjective welfare) “welfare”, “economic security”, “capability for good health”, “access to what I need”, “opportunity to do what I want”.

### 6.2. Results

Principal component analysis (PCA) extracted four factors with Eigenvalues > 1 (*KMO* = 0.94, *Chi*^2^ = 10,636.91). Factor scores for the Varimax rotated factors are listed in Table 2.

Whereas meaning in life and psychological richness load mainly on separate factors, happiness (four out of five items) and subjective welfare (all items) load on the same factor. Therefore, we ran a new, separate, PCA of the ten happiness and subjective welfare items, see Table 3.

Two factors with Eigenvalues > 1 were extracted (*KMO* = 0.91, *Chi*^2^ = 3466.34). All five happiness items loaded on the first factor and all five subjective welfare items loaded on the second factor.

### 6.3. Discussion

All taken together, the three wellbeing dimensions seem to have distinctly unique properties, whereas happiness and subjective welfare overlap somewhat. However, when only happiness and subjective welfare are analyzed, they load on distinct factors.

To further explore people’s assessments of wellbeing and subjective welfare, we put them in the context of living a good life, which is the focus of Study 3.

## 7. Study 3: How Do Wellbeing and Subjective Welfare Contribute to a Good Life?

In this study, we explored how wellbeing and subjective welfare contribute to a good life by entering people’s global ratings of their happiness, meaning in life, psychological richness, and subjective welfare, into a stepwise regression with an index measure of good life as the dependent variable.

### 7.1. Materials and Methods

A total of 710 Swedes (50% females, age span 18–70) were recruited via Facebook ads for a survey of wellbeing and welfare. They received the same information as in the previous studies.

For the independent measures, respondents answered the question, “how would you rate your life in terms of…” on a scale from 1 (very low)–10 (very high), with four global items: “happiness”, “meaning in life”, “psychological richness” and “welfare”. For the dependent measure good life, respondents rated their lives on the same scale with four items: “a good life”, “a life well-lived so far”, “a life worth living”, “a life I would wish for others”, *Cronbach’s alpha* = 0.90).

### 7.2. Results

The four independent variables were entered into a stepwise forward regression (*p* < 0.05) with the good life index as a dependent variable. The regression results are listed in Table 4.

In step 1, happiness was entered as the main predictor of good life ratings. In the next step, subjective welfare was entered as the second predictor. Meaning in life was entered as the third predictor. At *p* = 0.058, psychological richness was not entered into the equation.

### 7.3. Discussion

This study finds that the (two out of three) wellbeing dimensions and subjective welfare contribute significantly and uniquely to people’s assessments of living a good life. We used global measures, and people rated their wellbeing and subjective welfare in absolute terms. However, as discussed previously, research suggests that people may assess dimensions of their welfare in relative terms. Therefore, we conducted an additional study where people were asked to rate their wellbeing and subjective welfare globally in relative terms vis à vis the median.

## 8. Study 4: How Do Relative Wellbeing and Subjective Welfare Contribute to a Good Life?

In this study, we explored how people’s ratings of their relative wellbeing and subjective welfare contribute to a good life. People’s relative ratings vis à vis the median of their global happiness, meaning in life, psychological richness and subjective welfare were entered into a stepwise regression with an index measure of good life as the dependent variable.

### 8.1. Materials and Methods

A total of 663 Swedes (51% females, age span 18–70) were recruited via Facebook ads for a survey of wellbeing and welfare. They received the same information as in the previous studies.

For the independent measures, respondents answered the question, “compared to the median, how you rate your life in terms of…” on an 11-point scale from −5 (far below the median) to +5 = (far above the median) with four global items: “happiness”, “meaning in life”, “psychological richness” and “welfare”. For the dependent measure, we used the same scale from 1 (very low)–10 (very high) and items (“a good life”, “a life well-lived so far”, “a life worth living”, “a life I would wish for others”, *Cronbach’s alpha* = 0.91) as in Study 3.

### 8.2. Results

The four independent variables were entered into a stepwise forward regression (*p* < 0.05) with the good life index as a dependent variable. The regression results are listed in Table 5.

In step 1, happiness was entered as the main predictor of good life ratings. In the next step, subjective welfare was entered as the second predictor. Meaning in life was entered as the third predictor. Psychological richness was entered as the fourth predictor.

### 8.3. Discussion

Results were similar to Study 3. When assessed in relative terms vis à vis the median, the wellbeing dimensions and subjective welfare contribute significantly and uniquely to people’s assessments of living a good life.

## 9. Discussion

This paper set out to explore whether people are able to rate the importance of wellbeing and welfare in their lives, and how their assessments of wellbeing and welfare relate and contribute to their experience of living a good life. To this end, we conducted four online surveys.

The first study found that individuals were able to explicitly rate the importance of three dimensions of their wellbeing, and of their own welfare, and that all four ratings were significantly different from each other and only moderately overlapping. The second study found that, taken together, the three wellbeing dimensions load mainly on three unique factors, whereas happiness and subjective welfare loaded on the same factor. However, when subject to a separate two-factor analysis, happiness and subjective welfare loaded on separate factors. The third study found that people’s global assessments of their wellbeing in terms of happiness and meaning in life (but only marginally, psychological richness), and of their welfare, contribute to their experience of living a good life. In the fourth study, people assessed their global wellbeing and own welfare in relative terms vis à vis the median, and it was found that all three wellbeing dimensions and welfare contribute to their experience of living a good life.

Throughout the studies, we explored subjective welfare as a potential new construct, which would tap directly into individuals’ perceptions of their own welfare, separately from wellbeing. Measured globally, both in absolute and relative terms, subjective welfare did indeed contribute uniquely and significantly to individuals’ perceptions of living a good life. This is a first indication that people would be able to rate their own subjective welfare and differentiate it from wellbeing. We also drew adjectives from the capabilities- and needs-and-wants approaches to welfare to create a five-item measure. While the five items loaded on the same factor, their nomological properties in relation to Oishi et al.’s [22] happiness measure were far from perfect. More work is needed on the nomological network of wellbeing and subjective welfare, as well as the adjectives associated with subjective welfare.

In the final two studies, we also explored a quantitative global measure of good life. The four-item measure had a high alpha and discriminated well between the wellbeing dimensions, and subjective welfare, as a dependent variable. To the best of our knowledge, this is a first quantitative test of good life as a higher order construct, under which all dimensions of wellbeing, and subjective welfare, could potentially be sorted.

## 10. Conclusions

We believe that the constructs of subjective welfare and good life would be beneficial to public policy in future efforts to increase both wellbeing and welfare, per the UNs’ sustainable development goals [1]. They could potentially aid both policy makers, and aid and empower individuals, in prioritizing and making trade-offs between wellbeing and welfare when necessary [42]. It is also our hope that they would be a small contribution toward solving the conceptual confusion pertaining to wellbeing and welfare.

It is important to note that the studies in this paper are purely exploratory. They only included Swedish respondents, and they were recruited via Facebook ads through self-selection. For database reasons, we were not able to collect personal demographic information. For replication, future research needs to widen the scope and context of individuals cross-culturally, and to include probability sampling beyond facebook for external validity. The psychometric properties of the subjective welfare and good life measures need testing, as well as alternative operationalizations. As a next step, the nomological network should be widened to include antecedents, mediators and moderators, and their differential effects, to learn more about how wellbeing and subjective welfare can best be prioritized dependent on means and circumstances.

## Figures and Tables

**Table 1:** Descriptive statistics and correlations for individuals’ importance ratings of wellbeing and subjective welfare.

	*n*	*M*	*SD*	1.	2.	3.	4.
1. Happiness	707	7.68 ^b^	1.78	-			
2. Meaning in life	702	7.36 ^c^	1.76	0.29 ***	-		
3. Psychological richness	701	6.46 ^d^	1.95	0.052	0.24 ***	-	
4. Subjective Welfare	701	8.24 ^a^	1.69	0.39 ***	0.28 ***	0.14 ***	-

Note: *** *p* < 0.001. *a* > *b*, *t* = 7.67 *p* < 0.001. *b* > *c*, *t* = 4.03 *p* < 0.001. c > *d*, *t* = 10. 56 *p* < 0.001.

**Table 2:** Factor loadings for the three wellbeing dimensions and subjective welfare.

	Factor 1	Factor 2	Factor 3	Factor 4
***Happiness items***				
Happy	0.46	**0.68**	0.018	−0.035
Pleasant	**0.67**	0.45	−0.078	0.046
Simple	**0.61**	0.20	−0.29	0.091
Comfortable	**0.75**	0.19	−0.070	0.026
Stable	**0.62**	0.32	−0.28	0.093
***Meaningfulness items***				
Meaningful	0.29	**0.84**	0.083	0.063
Fulfilling	0.31	**0.85**	0.13	0.040
Purposeful	0.17	**0.81**	0.086	0.13
Dedicated	0.17	**0.78**	0.18	0.24
Virtuous	0.15	0.17	0.050	**0.94**
***Psychological richness items***				
Psychologically rich	0.20	**0.60**	0.35	−0.043
Eventful	0.22	0.48	**0.66**	−0.098
Interesting	0.30	**0.62**	0.49	−0.014
Dramatic	−0.16	0.002	**0.86**	0.058
Surprising	0.013	0.30	**0.80**	0.068
***Subjective Welfare items***				
Welfare	**0.76**	0.085	0.20	0.18
Economic security	**0.80**	0.086	0.070	0.060
Access to what I need	**0.75**	0.25	0.078	0.015
Capability for good health	**0.71**	0.25	0.056	0.013
Opportunity to do what I want	**0.66**	0.27	0.15	−0.084

Note: Factor loadings > 0.5 in bold.

**Table 3:** Factor loadings for happiness and subjective welfare.

	Factor 1	Factor 2
***Happiness items***		
Happy	**0.65**	0.35
Pleasant	**0.80**	0.34
Simple	**0.83**	0.089
Comfortable	**0.67**	0.41
Stable	**0.61**	0.39
***Subjective Welfare items***		
Welfare	0.19	**0.85**
Economic security	0.24	**0.85**
Access to what I need	0.41	**0.71**
Capability for good health	0.48	**0.59**
Opportunity to do what I want	0.48	**0.53**

Note: Factor loadings > 0.5 in bold.

**Table 4:** Stepwise regression of a good life on the three wellbeing dimensions and subjective welfare.

	Step 1	Step 2	Step 3	Step 4
Happiness	0.73 (0.027) ***	0.58 (0.030) ***	0.43 (0.036) ***	
Meaning in life	-	-	0.22 (0.033) ***	
Psychological richness	-	-	-	
Subjective Welfare	-	0.26 (0.028) ***	0.23 (0.027) ***	
*R* ^2^	0.51	0.57	0.59	-
Adjusted *R*^2^	0.51	0.57	0.59	-
Δ*F*	744.29 ***	89.12 ***	46.08 ***	-

Note: *** *p* < 0.001.

**Table 5:** Stepwise regression of a good life on the three wellbeing dimensions and subjective welfare (relative measures).

	Step 1	Step 2	Step 3	Step 4
Happiness	0.56 (0.027) ***	0.48 (0.030) ***	0.34 (0.041) ***	0.37 (0.042) ***
Meaning in life	-	-	0.19 (0.040) ***	0.24 (0.042) ***
Psychological richness	-	-	-	−0.12 (0.036) ***
Subjective Welfare	-	0.18 (0.030) ***	0.16 (0.030) ***	0.16 (0.030) ***
*R* ^2^	0.41	0.44	0.46	0.47
**Adjusted *R*^2^**	0.41	0.44	0.45	0.46
**Δ** ***F***	451.48 ***	36.38 ***	28.56 ***	11.14 ***

Note: *** *p* < 0.001.

## Data Availability

The data presented in this study are openly available in OSF at DOI 10.17605/OSF.IO/VQFSW.
